# Collection of datasets with DNS over HTTPS traffic

**DOI:** 10.1016/j.dib.2022.108310

**Published:** 2022-05-27

**Authors:** Kamil Jeřábek, Karel Hynek, Tomáš Čejka, Ondřej Ryšavý

**Affiliations:** a Faculty of Information Technology BUT, Bozetechova 1/2, Brno 612 00, Czech Republic; b Faculty of Information Technology CTU, Thakurova 9, Prague 160 00, Czech Republic; c CESNET a.l.e, Zikova 4, Prague 160 00, Czech Republic

**Keywords:** DNS over HTTPS, DNS, HTTPS, Computer, Network, Monitoring, Network traffic

## Abstract

Recently, the Internet has adopted the DNS over HTTPS (DoH) resolution mechanism for privacy-aware network applications. As DoH becomes more disseminated, it has also become a network monitoring research topic. For comprehensive evaluation and comparison of developed classifiers, real-world datasets are needed, motivating this contribution. We created a new large-scale collection of datasets consisting of two classes of traffic: i) DoH HTTPS communication and ii) non-DoH HTTPS connections. The DoH traffic is captured for multiple DoH providers and clients to include nuances of various DoH implementations and configurations. The non-DoH HTTPS connections complement the DoH communication aiming to include a wide range of existing network applications. The dataset collection consists of network traffic generated in a controlled environment and traffic captured from a real ISP network. The resulting datasets thus provide real-world network traffic data suitable for evaluating existing classifiers and the development of new methods.

**Specifications Table**

**Table:** 

Subject	Computer Networks and Communications
Specific subject area	Monitoring of encrypted network traffic, its analysis and classification
Type of data	Packet captures in the form of PCAP files and CSV files with flow data enriched for TLS metadata such as Server Names (from TLS SNI extensions), JA3 fingerprint, and used application protocol (from TLS ALPN extension)
How data were acquired	There are two types of datasets – *(i)* Generated, and *(ii)* Real-world. The *generated* datasets was obtained by generating traffic by DoH enabled web browsers towards multiple DoH resolvers. The *real-world dataset* was obtained by capturing at the perimeter CESNET2 network, a large internet service provider network type with around half a million users.
Data format	Raw (PCAP files) and Analyzed (CSV files)
Description of data collection	Datasets were collected using a tcpdump packet capture program locally on computer hosts or on the monitoring points located at the perimeter of the CESNET2 network. The CESNET2 capturing was performed with packet filtering based on 246 IP addresses of known DNS over HTTPS resolvers and port 443. The non-DoH traffic was captured based on IP filtering of address space assigned to university campus located in the Czech Republic. Packet capturing was performed on each monitoring point separately. The partial captures were then merged together and anonymized. The anonymization hides actual MAC addresses, transferred payload, and IP addresses of the DoH Clients. The IP addresses of DoH resolvers were left intact.
Data source location	Brno University of Technology, Brno, Czech Republic. CESNET z.s.p.o, Prague, Czech Republic.
Data accessibility	Repository name: Zenodo
	Data identification numbers: [10.5281/zenodo.5957277, 10.5281/zenodo.5957121, 10.5281/zenodo.5957420, 10.5281/zenodo.5957465, 10.5281/zenodo.5957676, 10.5281/zenodo.5957659, 10.5281/zenodo.5956043, 10.5281/zenodo.6024913]
Direct links to dataset parts:	**DoH-Gen-F-AABBC** — https://doi.org/10.5281/zenodo.5957277 **DoH-Gen-F-FGHOQS** — https://doi.org/10.5281/zenodo.5957121 **DoH-Gen-F-CCDDD** — https://doi.org/10.5281/zenodo.5957420 **DoH-Gen-C-AABBCC** — https://doi.org/10.5281/zenodo.5957465 **DoH-Gen-C-DDD** — https://doi.org/10.5281/zenodo.5957676 **DoH-Gen-C-CFGHOQS** — https://doi.org/10.5281/zenodo.5957659 **DoH-Real-world** — https://doi.org/10.5281/zenodo.5956043 **Supplementary files** — https://doi.org/10.5281/zenodo.6024913

## Value of the Data

• The datasets consist of captures of DNS over HTTPS (DoH) traffic and non-DoH HTTPS traffic that comes both from controlled and real-world environment. The real-world dataset was collected in an Internet Service Provider (ISP) backbone network servicing half-million users. DoH captures consist of traffic generated by several techniques and clients towards 16 selected DoH servers of various implementations and with different configurations. The generated part contains 64,000 web page accesses and related DoH communication. The datasets aim to provide a comprehensive sample of DoH traffic as observed in real networks and various available implementations and configurations.
• Researchers can use the provided collection as i) a benchmark for the DNS over HTTPS protocol classification and detection algorithms [1], [2], [3], ii) for studying differences between the behavior and performance of various DNS resolution methods [4], [5], and because of the presence of both DoH and HTTPS communication also for other iii) encrypted traffic analysis research [6], [7]. We redacted the real-world data by anonymization of IP addresses, preserving all other features of communication. Moreover, we also included domain names from TLS headers to allow TLS traffic classification [8], [9].
• The datasets collection enables researchers to experiment with DNS over HTTPS traffic recognition and pattern analysis. However, since the data are provided in raw packet captures, it can be suitable for other network traffic analysis tasks, e.g., it can be also used as a real-world benign traffic sample in malware identification challenges [10], [11].
• The datasets provide a large and unique combination of labeled traffic. The generated DoH and labeled real-world HTTPS traffic provide the ground truth data for training network classifiers and evaluating their performance. Moreover, packet captures can be used for IP Flow-based traffic analysis, detection, and classification to experiment with novel traffic features.
• The datasets collection can also improve the understanding of DoH behavior and other HTTPS traffic phenomena in a large-scale network environment. It is possible to observe and analyze performance and other relevant metrics to improve the specification and implementations.
• We are not aware of any other dataset of the comparable size and variety that provides real HTTPS traffic captured on a large-scale ISP in the form of raw packet captures that enable unbounded extraction of arbitrary available features.

## 1 Data Description

The datasets consist of labeled HTTPS traffic. The traffic is either the DoH or non-DoH HTTPS communication. The primary focus of this dataset collection is to provide the most variable and representative DoH traffic obtained from communication with many available DoH services. To allow comparison and discrimination of DoH characteristics with other HTTPS traffic types, we also provide general HTTPS traffic captures. Moreover, DoH and general HTTPS traffic comprehensively cover web traffic, allowing dataset utilization for other research tasks not limited to the DoH area.

**Fig. 1:**
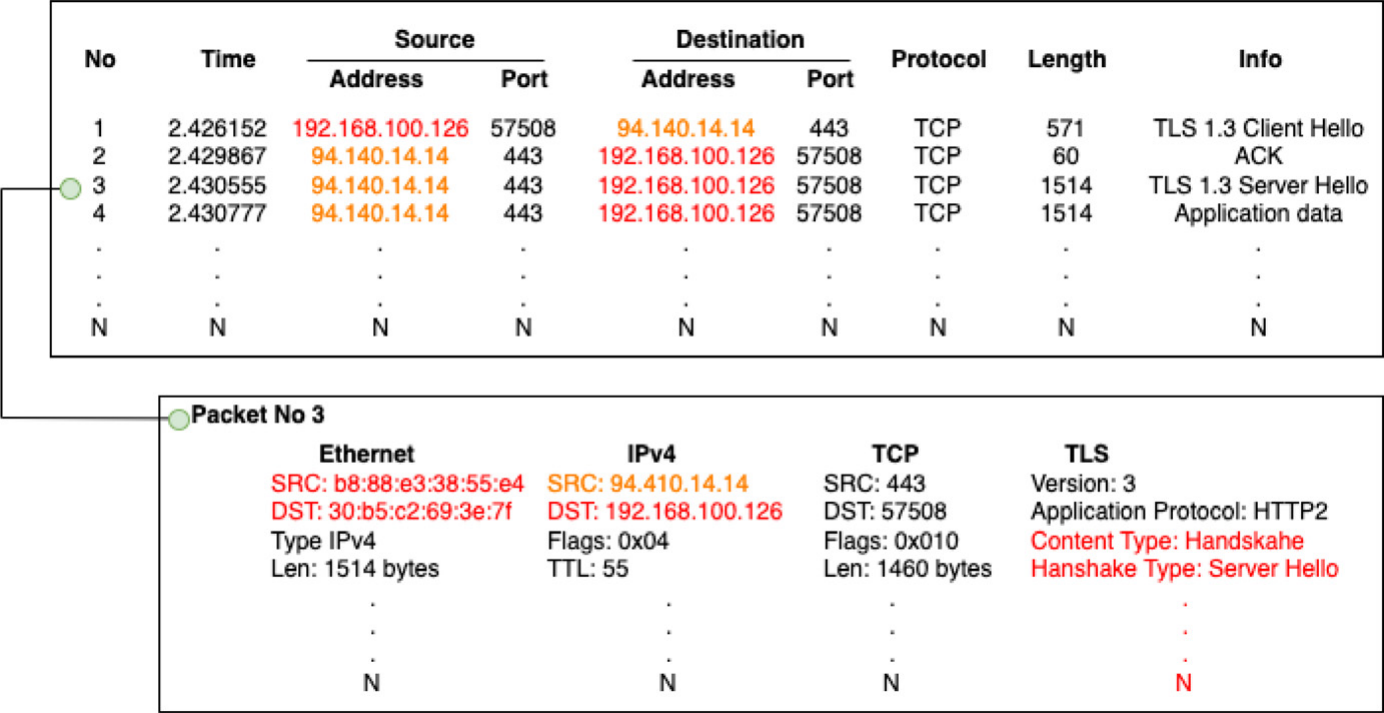
Example of captured network traffic samples. The font color indicate field anonymization in *real-world* data, the generated data are not anonymized. The fields that can be extracted in original form are denoted in **black**, anonymized values are denoted in **red**, and **orange** color denotes fields which were left intact, since they belong to known DoH resolver. (For interpretation of the references to color in this figure legend, the reader is referred to the web version of this article.)

The collection of datasets contains DoH and HTTPS packets in the total amount of 430 GB raw binary data. The datasets were captured in two enviroments — (i) *generated* DoH and HTTPS traffic from a controlled experimental system and (ii) *real-world* DoH and HTTPS traffic from a real large ISP network. The aim of generated data is to provide heterogeneous DoH traffic for various existing DoH implementations. Real-world captured data reflects the real network environment, e.g., packet timing, lost and retransmissions, and connection errors. Full-packet captures are in standardized PCAP format [12], which is a default for libpcap library and broadly supported by network analysis software, e.g., Wireshark^1^, tcpdump^2^ or IDS Suricata.^3^ Full-packet captures can be considered the primary source of raw network data that can be used for further analysis or feature extraction. Fig. 1 exemplifies how various network features can be extracted from raw packets for network analysis.

In addition to raw packet captures, we also provide CSV representations of flow data enriched with information from TLS handshake. The flow data were generated for all captures (even the generated ones for dataset consistency). It contains server names (TLS SNI extension), used application protocol (TLS ALPN extension), and TLS JA3 fingerprints.^4^ All of these values are commonly used in encrypted traffic analysis [7], [13], [14]. The description of provided flow data fields is in Table 1.

**Table 1:** The description of column headers in CSV files with extended flow data.

Column Name	Column Description
DST_IP	Destination IP address
SRC_IP	Source IP address
BYTES	The number of transmitted bytes from Source to Destination
BYTES_REV	The number of transmitted bytes from Destination to Source
TIME_FIRST	Timestamp of the first packet in the flow in format YYYY-MM-DDTHH-MM-SS
TIME_LAST	Timestamp of the last packet in the flow in format YYYY-MM-DDTHH-MM-SS
PACKETS	The number of packets transmitted from Source to Destination
PACKETS_REV	The number of packets transmitted from Destination to Source
DST_PORT	Destination port
SRC_PORT	Source port
PROTOCOL	The number of transport protocol
TCP_FLAGS	Logic OR across all TCP flags in the packets transmitted from Source to Destination
TCP_FLAGS_REV	Logic OR across all TCP flags in the packets transmitted from Destination to Source
TLS_ALPN	The Value of Application Protocol Negotiation Extension sent from Server
TLS_JA3	The JA3 fingerprint
TLS_SNI	The value of Server Name Indication Extension sent by Client

### 1.1 Generated data

The datasets with generated traffic were created in a controlled environment to provide samples of DoH communication for different existing implementations in operating systems and web browsers. The traffic originates in DoH-capable browsers Firefox and Chrome. Browsers access a large collection of web pages and both DoH and HTTPS communication are captured. Table 2 lists of generated pcap files.

**Table 2:** Properties of Generated datasets. Abbrevation stands for: **Brws** — Used Browser, **F** — Firefox, **C** — Chrome.

Name	DoI	Brws	Resolvers
DoH-Gen-F-AABBC	10.5281/zenodo.5957277	F	AdGuard, AhaDNS, BlahDNS, BraveDNS, CloudFlare
DoH-Gen-F-CCDDD	10.5281/zenodo.5957420	F	Comcast, CZNIC, DNSForge, DNSSB, DOHli
DoH-Gen-F-FGHOQS	10.5281/zenodo.5957121	F	FFMuc, Google, Hostux, OpenDNS, Quad9, Switch
DoH-Gen-C-AABBCC	10.5281/zenodo.5957465	C	AdGuard, AhaDNS, BlahDNS, BraveDNS, Comcast, CZNIC
DoH-Gen-C-DDD	10.5281/zenodo.5957676	C	DNSForge, DSNSB, DOHli
DoH-Gen-C-CFGHOQS	10.5281/zenodo.5957659	C	CloudFlare, FFMuc, Google, Hostux, OpenDNS, Quad9, Switch

The captured files contain a mix of non-DoH HTTPS and DoH traffic. To discriminate the DoH traffic, we provide with each dataset a list of IP addresses of known DoH resolvers used in our experiments. This list can be used to identify and thus label the DoH flows in the PCAP files.

A sample of 16 different DoH servers was chosen to include a diversity of traffic characteristics related to domain name resolution. For each DoH server, we generated traffic by fetching 2000 websites on each web browser. Totally, 64,000 websites requests were generated and corresponding HTTPS communication was captured in the dataset. Firefox web browser supports both GET and POST methods for resolving DoH, therefore 1000 websites were visited by using the DoH GET method, and the other 1000 websites access were performed with the DoH POST method. Note that traffic samples generated by the Firefox browser contain packets up to 64 KB because the browser was executed in a virtual environment with TCP offloading enabled [15]. We decided to include it in the datasets as this is one of the valid real-world scenarios.

Datasets with Firefox data have captures located in the /data/generated/pcap/firefox/ directory. Each filename consists of the HTTP method involved in resolution (GET, POST) and the name of the DoH server used for resolution. Table 3a shows summary metrics of all files created by Firefox across all generated datasets in the collection.

**Table 3:** Total statistics of Firefox and Chrome generated data.

Total statistics of Firefox generated data.	
Name	Value
Total Data Size	131 GB
Total files	32
DoH connections	∼255 K
Non-DoH connections	∼995 K

Total statistics of Chrome generated data.	

Total Data Size	119.5 GB
Total files	32
DoH connections	∼91 K
Non-DoH connections	∼634 K

**Fig. 2:**
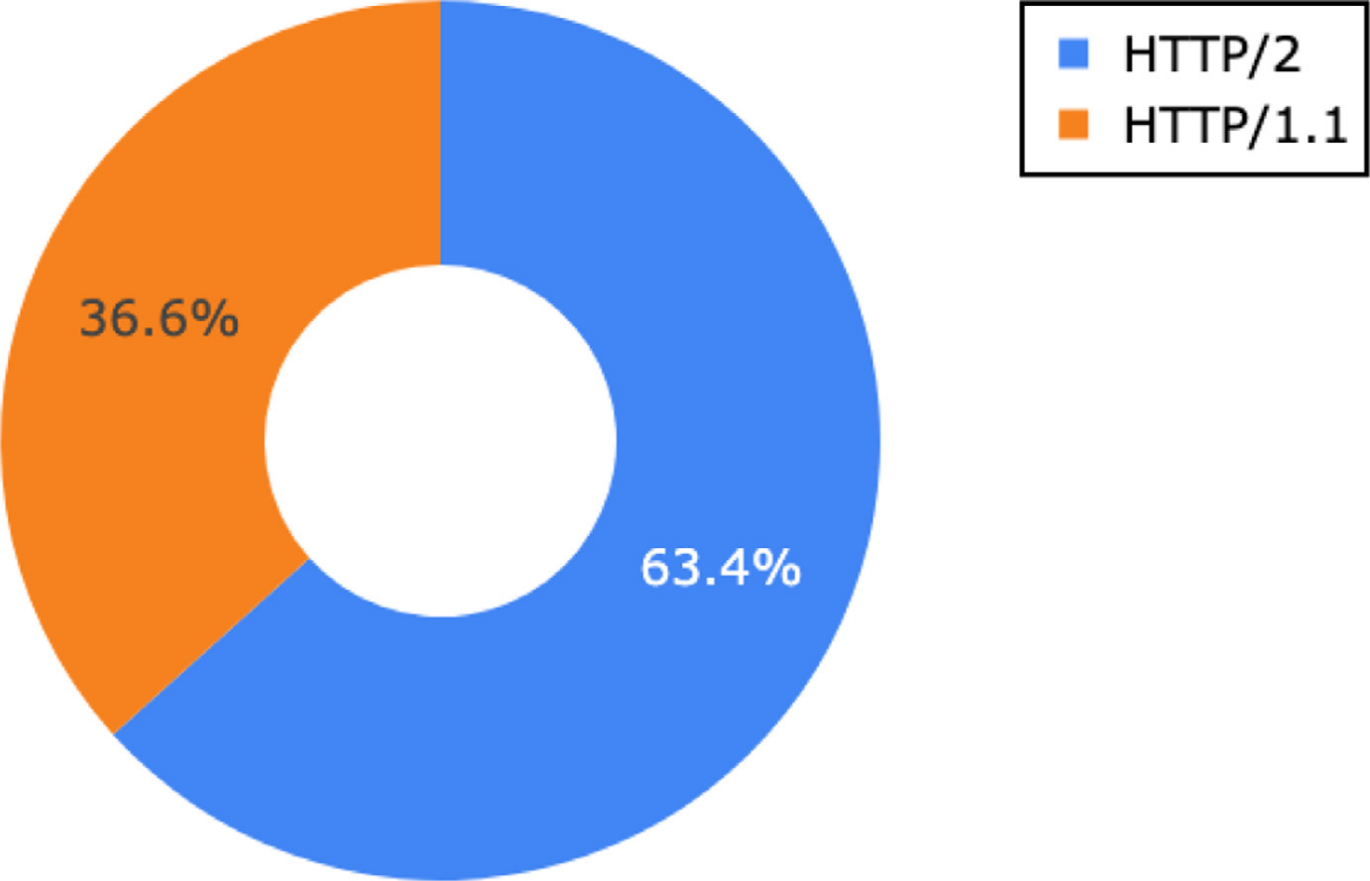
Ratios of application layer protocols extracted from APLN extension in generated TLS traffic (both Firefox and Chrome).

The datasets generated using by Google Chrome are all located at generated/pcap/chrome/. Each subdirectory name determines used DoH server. Table 3b depicts summary information about all files created with Chrome browser across all generated datasets in the collection. The difference in the number of connections can be caused by the generating process discussed later and browser-specific implementation. The ratio between used HTTP protocol version in generated datasets is shown in Fig. 2.

The Chrome browser implements EDNS padding, slightly impacting the DoH flow characteristics when compared to the Firefox DoH flow which yields an increased diversity of DoH traffic.

### 1.2 Real-world data

The *real-world* data are provided in the DoH-Real-world dataset (10.5281/zenodo.5956043) and contains DoH HTTPS and web-based HTTPS communication. The real-world traffic captures were anonymized to protect the privacy of real users (packets have no payload, clients IP, and MAC addresses were hashed). However, the anonymization does not pose a major limitation for data usability, since it maintains original timestamps of the packets, and other important network traffic characteristics.

The DoH captures contain only DNS over HTTPS communication, obtained by filtering connections using the IP addresses of known DoH resolvers. The list of known DoH resolvers was taken from [16] and it is included within the dataset, and also in the supplementary files, see Section 1.3. The used list of DoH resolvers’ IP addresses remained unchanged for all DoH traffic captures.

The HTTPS traffic captures contain all communication transmitted over standard HTTPS port TCP/443, including DoH since DNS over HTTPS also runs on standard HTTPS port. Nevertheless, the provided list of identified DoH addresses can be used to discriminate DoH from non-DoH traffic. The complete list of packet capture files is written in Tables 4 and 5. The packet capturing was performed between June and October 2021 for several days. Packet captures do not overlap.

**Table 4:** Real-world DoH captures statistics.

File Name (pcap)	Size	Date of capture	Dur.	Conn.	Packets
DoH-01082021-48h	6 GB	2021-08-01	48h	435,313	29 M
DoH-03082021-48h	5 GB	2021-08-03	48h	329,974	22 M
DoH-06102021-48h	19 GB	2021-10-06	48h	1,376,067	88 M
DoH-08102021-48h	8 GB	2021-10-08	48h	467,328	41 M
DoH-13072021-48h	5 GB	2021-07-13	48h	414,085	21 M
DoH-15072021-48h	4 GB	2021-07-15	48h	367,761	18 M
DoH-17072021-48h	3 GB	2021-07-17	48h	265,145	12 M
DoH-19072021-48h	5 GB	2021-07-19	48h	520,766	24 M
DoH-27072021-48h	6 GB	2021-07-27	48h	483,405	30 M
DoH-28062021-24h	3 GB	2021-06-28	24h	359,275	17 M
DoH-30072021-48h	3 GB	2021-07-30	48h	228,895	14 M

**Table 5:** Real-world HTTPS captures statistics.

File Name (pcap)	Size	Date of capture	Dur.	Conn.	Packets
HTTPS-20102021-10h	20 GB	2021-10-20	10h	43,616	17 M
HTTPS-20102021-12h	20 GB	2021-10-20	12h	33,864	18 M
HTTPS-21102021-12h	20 GB	2021-10-21	12h	33,993	17 M
HTTPS-04102021-01h-1	20 GB	2021-10-04	0.1h	15,028	17 M
HTTPS-04102021-01h-2	20 GB	2021-10-04	0.1h	16,124	17 M
HTTPS-04102021-02h	20 GB	2021-10-04	0.2h	13,410	18 M

**Fig. 3:**
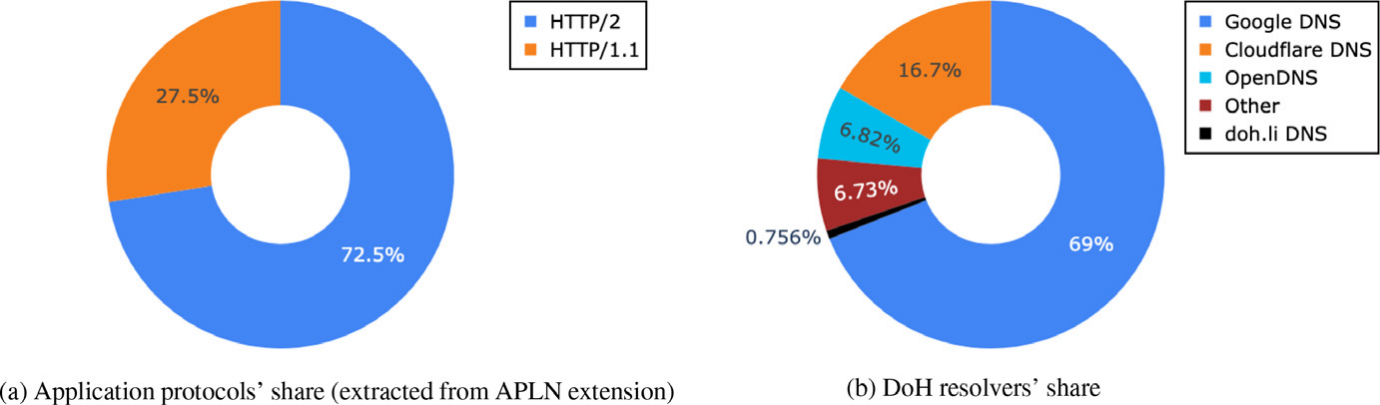
The share of DoH resolvers and application protocols presented in the real-word dataset.

Contrary to the *generated* traffic, the DoH communication was captured in the real network, which reflects ratios of most utilized DoH Internet services as seen in Fig. 3. The most DoH connections involve Google DoH resolvers, and the five most popular resolvers represent more than 93% of all DoH traffic.

The importance of the *real-world* dataset is that it contains the HTTPS traffic of thousands of genuine users. The Table 6 shows overall statistics of captured dataset. It should be noticed that there is 116,263 unique client addresses in the network. Since the capture was created on an Internet Service Provider (ISP) backbone, each IP address may correspond to multiple physical/virtual devices due to Network Address Translation (NAT). The actual number of clients can thus be larger.

**Table 6:** Total stats of captured data.

Name	Value
Total Data Size	179 GB
Total Time	∼10 Days
Connections	∼420 M
Number of unique Client IP addresses	116,263
Number of unique Server IP addresses	9343
Number of unique DoH Resolver’s IP addresses	142

### 1.3 Supplementary files

In addition to the main dataset content, we also provide Supplementary files (10.5281/zenodo.6024913) consisting of software scripts for data generation and post-processing. The list and short description of supplementary files, are provided in Table 7.

**Table 7:** The list and description of supplementary files.

Name	Description
doh-resolvers	The list of IP addresses of known DoH resolver, which was used during the *real-world* part creation. The list is in the CSV file format.
pcap-anonymizer	Python scripts used for the anonymization of real captures.
firefox|chrome/scripts/	Folder containing scripts and other files used for data generation.
firefox|chrome/domains/<num>.csv	Files containing the domains used for data generation.
doh_server_urls.txt	File containing DoH server urls used in generation process.

## 2 Experimental Design, Materials and Methods

In this section, we describe the data acquisition environments. Firstly, we describe the environment used for DoH traffic generation (Section 2.1), then we provide an overview of *Real-World Data* acquisition procedure and anonymization (Section 2.2).

The datasets contain all DoH and non-DoH captured HTTPS flows. The common attribute of the whole dataset collection is that the data are captured in a pure form, where the process and all necessary information are covered in this paper. Hence, the dataset may contain all sorts of DoH and non-DoH HTTPS flows, e.g., long, short, successful, and failed in the way they appeared during the capturing process. We have not performed any additional filtering that would lead to information loss except those mentioned in this section.

### 2.1 Generated data

Two DoH-enabled web browsers were used for making web requests. We selected Chrome and Firefox because they have the most advanced DoH implementations. Since we primarily target DoH and Non-DoH traffic generation, we have to cover all possible settings that can affect the DoH traffic characteristics (such as packet sizes) generated by those applications.

Chrome browser is based on Chromium code-base that shares DoH resolution implementation with other Chromium-based browsers such as Edge^5^, Brave.^6^

Firefox browser provides possibility of choosing DoH HTTP method, either GET or POST. It provides more management options than Chrome, such as whether to force DoH usage or not and which server to use (Cloudflare’s DoH service by default).

The *generated data* samples were created by visiting a collection of websites that are part of the Majestic million list [17]. The URLs were taken from the beginning of the list and each website was visited by one of the browsers. The list of visited websites is included in the supplementary files, see Section 1.3.

DoH traffic characteristics are mostly because of client-server interaction. DoH servers can employ different implementations and be deployed with different configurations. Moreover, the HTTPS ecosystem on the server-side can consist of proxies, firewalls, load-balancers, and other systems that impact network communication. To cover various DoH traffic patterns, we included 16 different DoH servers in our datasets. The 16 DoH servers were chosen to cover various DoH traffic patterns as described in [18], where we can find that the major difference is in the HTTP version and the number of HTTP headers used. The servers were taken from the same DoH monitoring tool as mentioned in the article [16], supporting different features, and each server was deployed by a different provider.

The CSV files with flow data enriched for TLS information were then created with ipfixprobe^7^ open-source flow exporter. We used its TLS plugin to create flow data extended for TLS information.

The whole traffic generation process took place at the Brno University of Technology, Faculty of Information Technology. The machines were directly connected to the university network.

#### 2.1.1 Firefox browser traffic generation

At first, the Firefox browser in version 76.0.1 was used in the Docker container. The use of the Docker container does not influence the traffic with specific properties; however, it simplified the generation process automation and provided an isolated application environment. The platform utilized in the data generation process using Firefox browser was Supermicro SuperTwin2 6026TT-TF server equipped with eight Intel (R) Xeon E5520 @ 2.26 GHz. The cluster consists of 4 nodes. The nodes were equipped with 48 GB RAM and 16 CPU cores. The platform was directly connected to the Brno University of Technology network. Network traffic was captured directly in the container using the tcpdump command-line tool.

Python and Selenium libraries were used to automate generation process. To be able to provide more realistic behavior of the browser, we used X-virtual frame buffer, which implements the X11 display server protocol where all graphical operations are made in virtual memory without graphical output [19]. The browser than works with a standard graphical output even within the container. In addition, we restricted the container to use 4 CPUs and 4 GB of RAM at maximum.

**Fig. 4:**
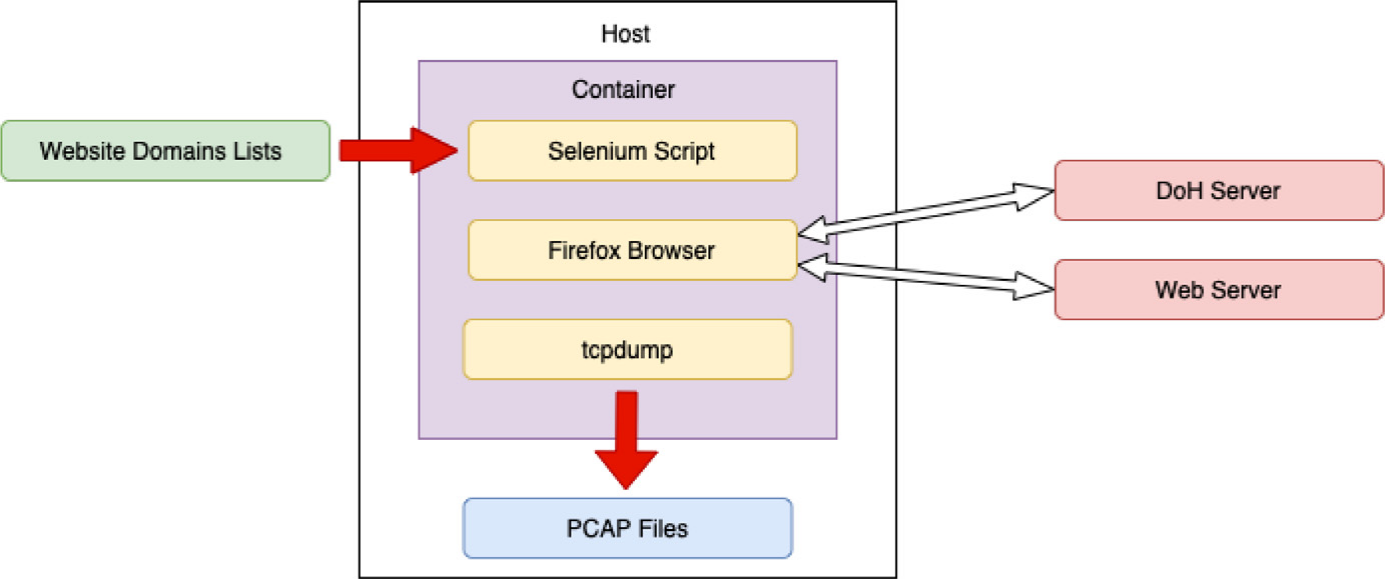
Firefox browser traffic generation schema.

Firefox had disabled the local web cache during web page access to get more network traffic samples. The process consists of opening the browser, fetching the website, timeout, and waiting for the loading of the website, followed by browser closing. The schema of this generation is depicted in Fig. 4. This process was repeated 1000 times for DoH POST and GET methods and for each of the 16 DoH servers. Together, 32,000 different websites were fetched.

#### 2.1.2 Chrome browser traffic generation

Since the used version of Chrome browser (94.0.4606.81) does not support DoH on Linux OS at the time of our data generation, we could not reuse the same generation environment as in the case of Firefox. The Chrome browser generation took place on a separate machine running Windows OS without Docker or any virtual environment, running directly on the bare metal. The machine was equipped with 3rd generation Intel Core i5, 8 GB RAM also connected directly to the same university network as in the case of Firefox.

**Fig. 5:**
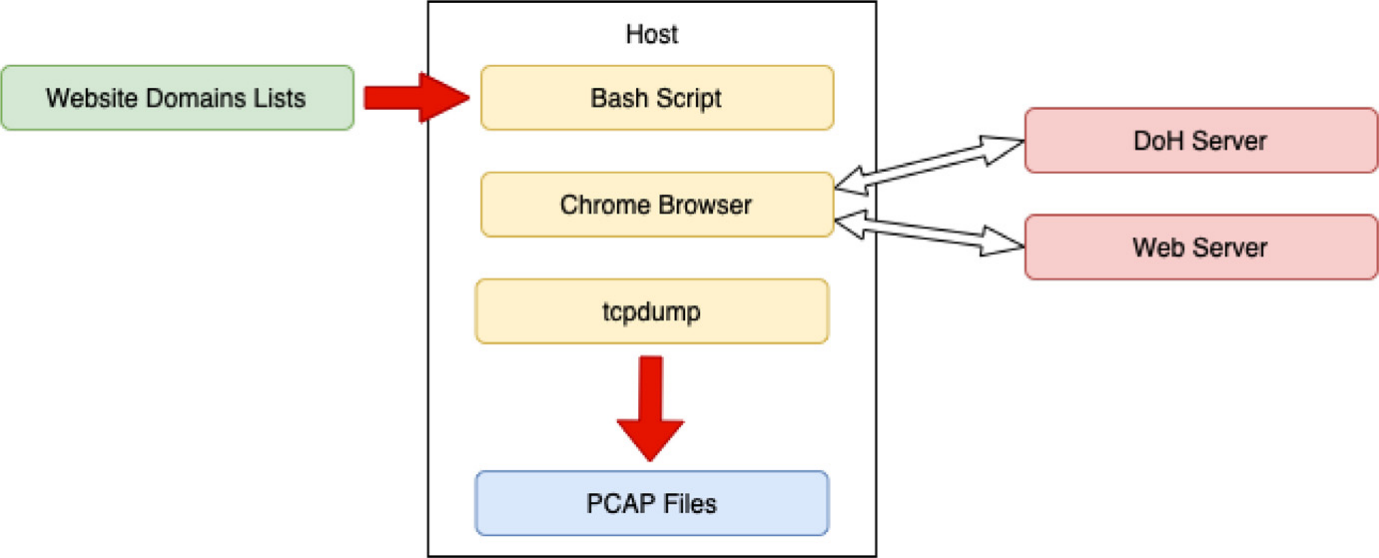
Chrome browser traffic generation schema.

The traffic generation process itself was similar to Firefox. It consisted of opening the browser with the given website, timeout, and waiting for loading the website, followed by browser closing. This generation is shown in supportive schema in Fig. 5. The automation was handled by a python script with a manual setting of custom DoH servers. The same 16 DoH servers as in the case of Firefox were used. Chrome does not allow users to set HTTP GET or POST methods for querying DoH. Hence, the amount of fetched websites is similar to the Firefox case: 2×1000 only with the default Chrome method used. In total, 32,000 different websites were fetched.

### 2.2 Real-World data

**Fig. 6:**
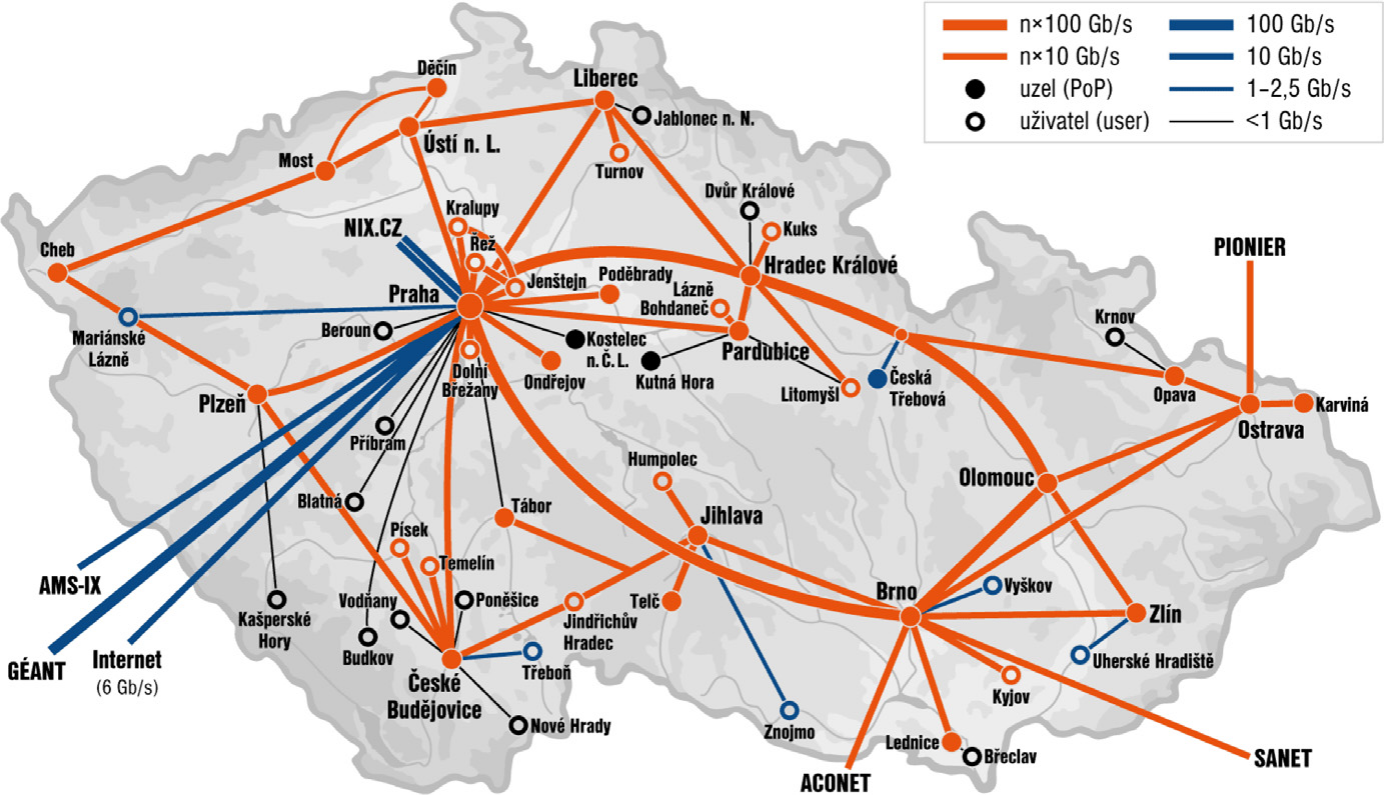
The topology of CESNET2 network.

The *Real-World data* of the dataset were captured on the monitoring points of the CESNET organization, which is the Czech National Research and Education Network operator. CESNET operates a backbone network infrastructure called CESNET2, which is used by half-million users. CESNET2 provides internet connectivity to universities, campuses, research centers, schools, hospitals, and some government offices. The topology of the CESNET2 backbone network is depicted at Fig. 6.

The six monitoring points are located at the perimeter of the CESNET2 network in three locations in the Czech Republic — Prague, Brno, and Ostrava. Each of them is connected to one or multiple 100 Gbps lines via passive optical TAP. Since the packet capture is distributed across multiple points and locations, we need to deal with time shifts on each monitoring server resulting in inaccuracies in packet timestamps.

The points are synchronized via Network Time Protocol (NTP), with an accuracy of up to 10 ms to minimize the time inaccuracies. The time shift between monitoring probes does not affect connections that are routed symmetrically, meaning that all packets belonging to the one TCP connection pass through the same monitoring point.

As a result, the Real-World Data contains inaccurate interarrival time values between packets in the opposite directions — packets in the same direction are always routed via the same monitoring points. According to long-term measurement in the CESNET2 network, 40 % of all 443/TCP connections are routed asymmetrically, thus affected by the time shifts. However, the timestamp inaccuracies are small, and they do not cause any significant disturbances in packet order within the connection (HTTPS requests are always before HTTPS responses). Moreover, since the distributed monitoring infrastructure is common in large-scale network monitoring [20], these data imperfections are viable for the dataset creating the detection algorithms prepared for such deployment.

**Fig. 7:**
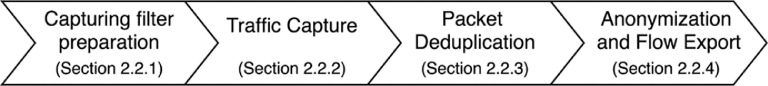
Real-World Data creation process.

As it can be seen in Fig. 7, the creation of Real-World data can be divided into four steps: *(i)* Capturing filter preparation, *(ii)* Traffic Capture, *(iii)* Deduplication, *(iv)* Anonymization. Each step is described in the following sections.

#### 2.2.1 Capturing filter preparation

The monitoring points can process 100 Gbps traffic; however, it is not possible to capture all ongoing traffic due to bandwidth limitation and writing speeds of the storage, which would result in missing packets in the dataset. Therefore, it was necessary to create a capturing filter with this limitation in mind. Since the use of DoH is still small, we could capture all DoH traffic by filtering based on the DoH resolver’s list (which is included with the dataset as supplementary file — described in Section 1.3).

In the case of HTTPS traffic, we have limited the capturing to the selected /24, and /22 IPv4 address ranges assigned to the university campus. Additionally, we have monitored the whole capturing process for packet drops to ensure that all transmitted packets in filtered connections were saved on the disks and the points were not overloaded; thus, no artificial packet drops were added.

Overall, we created two capturing filters, which remained unchanged for all traffic captures during the dataset’s creation. According to the monitoring of known DoH resolvers [16], there was not any change during the capturing period.

#### 2.2.2 Traffic capture

**Fig. 8:**
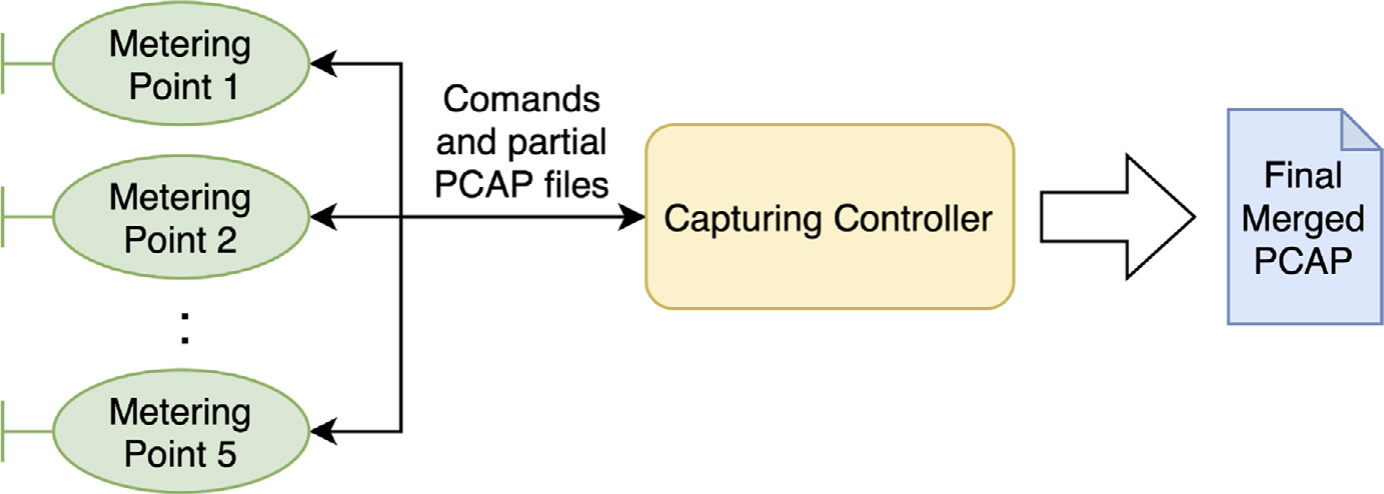
The schema of capture distribution.

Since the CESNET2 network is a large infrastructure with multiple monitoring points, we have created custom scripts for concurrent full-packet capture on each point using parallel SSH^8^. Fig. 8 shows a scheme of the overall capturing process. As the first step, the capturing controller distributes the command to the monitoring points. The command contains a filter and maximal duration of capturing. When the packet capture exceeds the maximum duration, the pcap file is distributed back to the controller. The controller then merges partial packet captures from the monitoring probes into a single one, using mergecap utility.^9^

#### 2.2.3 Packet deduplication

Since a backbone ISP network can also carry transit traffic, there is a chance of capturing duplicate packets on distributed monitoring infrastructure. The deduplication was performed with editcap^10^ utility with a time window of 10 ms. Thus, if there are two, or more duplicate packets within 10 ms time window, only the first packet will be maintained.

**Fig. 9:**
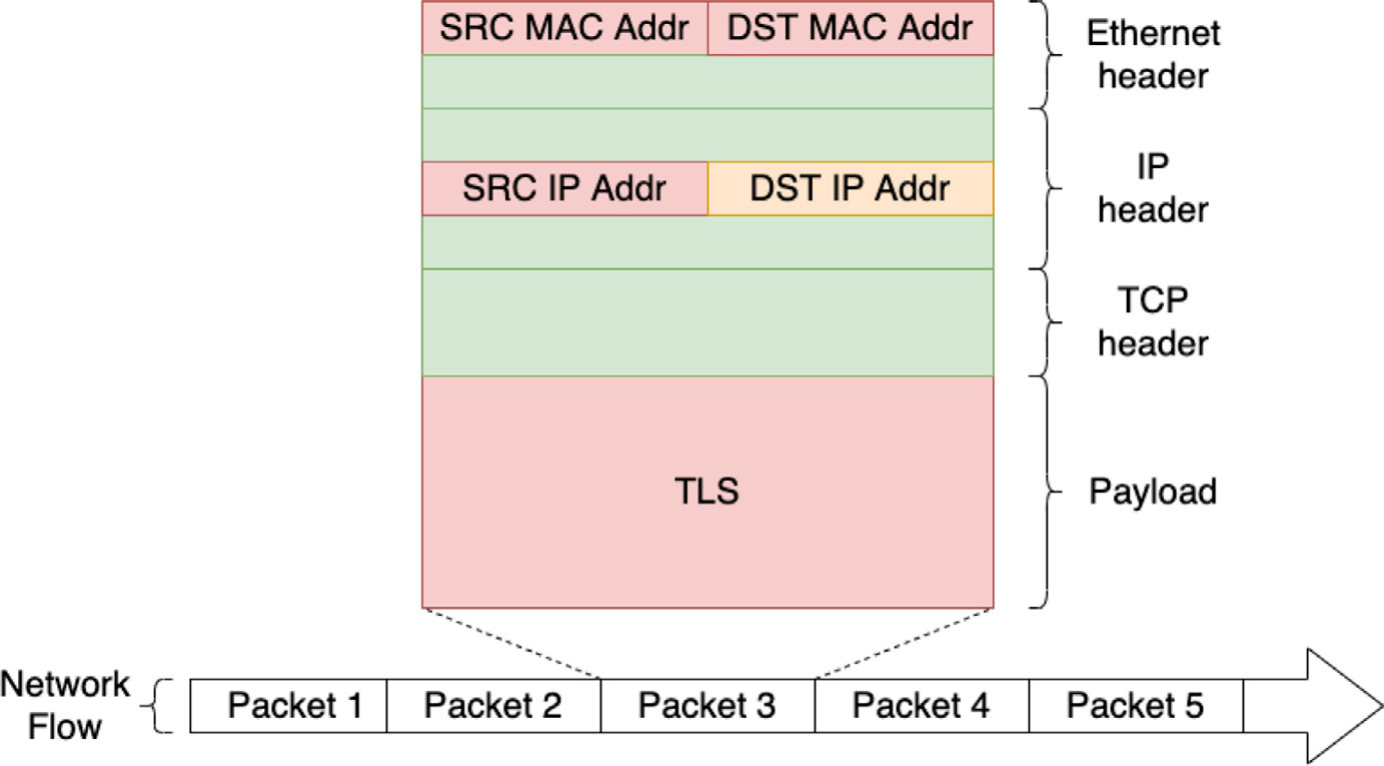
Structure of the packet. The **red fields** are always anonymized, **orange field** is anonymized for other IP addresses than the known DoH servers, and **green fields** are always left intact. (For interpretation of the references to color in this figure legend, the reader is referred to the web version of this article.)

#### 2.2.4 Anonymization and flow export

Since we are using data from real ISP backbone lines, we needed to remove sensitive information. The anonymization of captured traffic was performed automatically; thus, nobody could view or analyze raw source data. The packet fields, that were anonymized are shown within the packet context in Fig. 9.

At first, we anonymized all addresses (IPv4, IPv6, and MAC), except the IP addresses of DoH resolvers, which remained unmodified. The anonymization process replaced each address by a part of its SHA256 hash (the first N bytes, where N is the length of the corresponding address). The hashing algorithm ensures that a particular address is always mapped onto the same anonymized value.

PCAP files with anonymized IP addresses were then used to create TLS enriched flows with ipfixprobe flow exporter. The flows were created the same way as in the generated part.

After the flow export, we also anonymized the payload from all packets by substituting every byte with the letter ‘X’. Removing packets’ payload has only a negligible impact on the value of the data (since they are mostly encrypted anyway); however, it ensures the future privacy of the real users.

## Ethics Statement

Privacy of users is our essential priority, so our whole research was done with extreme carefulness. The indisputable advantages of real traffic generated by hundreds of thousands of people come with the cost of potential privacy abuse of real users. Therefore, we used only automatic data processing with immediate data anonymization. With this, we declare that we did not analyze or manually process deanonymized data, and we did not perform any procedures that could lead us to the user’s identity. Moreover, there is no possibility of revealing real users’ identities from the provided dataset.

## CRediT authorship contribution statement

**Kamil Jeřábek:** Writing – original draft, Conceptualization, Methodology, Software, Data curation. **Karel Hynek:** Writing – original draft, Conceptualization, Methodology, Software, Data curation. **Tomáš Čejka:** Writing – review & editing. **Ondřej Ryšavý:** Writing – review & editing.

## Declaration of Competing Interest

The authors declare that they have no known competing financial interests or personal relationships which have, or could be perceived to have, influenced the work reported in this article.

## Data Availability

DoH - Real-World (Original data) (Zenodo). DoH-Gen-C-CFGHOQS (Original data) (Zenodo). DoH-Gen-C-DDD (Original data) (Zenodo). DoH-Gen-C-AABBCC (Original data) (Zenodo). DoH-Gen-F-FGHOQS (Original data) (Zenodo). DoH-Gen-F-CCDDD (Original data) (Zenodo). DoH-Gen-F-AABBC (Original data) (Zenodo). Supplementary Material (Original data) (Zenodo).

## Footnotes

1 https://www.wireshark.com

2 https://www.tcpdump.org

3 https://suricata.io

4 https://github.com/salesforce/ja3

5 https://www.microsoft.com/en-us/edge

6 https://brave.com/

7 https://github.com/CESNET/ipfixprobe.git

8 https://linux.die.net/man/1/pssh

9 https://www.wireshark.org/docs/man-pages/mergecap.html

10 https://www.wireshark.org/docs/man-pages/editcap.html
